# Predictors of information needs among women with breast cancer receiving adjuvant therapy at Tikur Anbessa specialized hospital, Addis Ababa Ethiopia: a cross-sectional study

**DOI:** 10.1186/s12905-023-02805-2

**Published:** 2023-12-08

**Authors:** Sosina W. Tilahun, Leul D. Kitaw, Nete T. Yusuf

**Affiliations:** Department of Nursing, College of Health Sciences, Addis Ababa University, Addis Ababa, Ethiopia

**Keywords:** Information needs, Breast cancer, Adjuvant therapies

## Abstract

**Background:**

Women undergoing adjuvant therapy for breast cancer have diverse information needs that remain unfulfilled. Extensive research has shown that access to relevant information about their condition can significantly enhance the quality of life for these women, making it an essential part of cancer care. However, various clinical and socioeconomic factors influence the information needs of these women. Hence, the primary aim of this study is to identify predictors of the information needs of women undergoing adjuvant therapy for breast cancer. In addition, this study will also describe the preferred sources of information and the optimal timing for its acquisition.

**Methods:**

A facility-based cross-sectional study was undertaken at Tikur Anbessa Specialty Hospital, enlisting a cohort comprising 121 women undergoing adjuvant therapy for breast cancer. Trained interviewers administered an Amharic-translated Toronto information needs questionnaire specifically designed for breast cancer to assess the information needs of the study participants Statistical analysis was executed using the sophisticated software SPSS (version 25). Descriptive statistics were employed to summarize the variables of the study. A linear regression analyses was then carried out to identify notable predictors that significantly influenced the information needs of the women.

**Results:**

The total mean score for overall information needs in the current study was 194.30 (± 28.01), with a range scale of 142–260 and a standardized mean score of 3.74 (± 0.54). The disease and treatment domains had the highest information needs, with standardized mean scores (standard deviation) of 4.00 (± 0.54) and 3.77 (± 0.59), respectively. 95% of the participants sought information from healthcare professionals, and 67.7% of the women needed the information before beginning the treatments. Predictors of information needs were following a single treatment option (β = 12.68; 95% CI (0.68, 24.68); *P* = 0.039) and joining higher education and above (β = 17.1; 95% CI (1.47, 34.14); *P* = 0.033).

**Conclusion:**

The women exhibited a substantial demand for information. Healthcare professionals need to consider the women’s educational background and treatment status while delivering the needed information.

## Background

The breast holds significant meaning for women, embodying physical attributes as well as notions of femininity, allure, and sexuality [1, 2]. Nonetheless, breast cancer and its accompanying surgical interventions account for the majority of cases involving breast loss among women [2]. Surgery plays a pivotal role in addressing breast cancer, with the specific type of surgical procedure contingent upon considerations such as the stage of the cancer, tumor size, and patient preferences. The primary goals of surgery encompass eliminating the tumor, evaluating lymph node involvement, and mitigating the likelihood of recurrence [3, 4]. Supplementary therapy after surgical intervention, aimed at minimizing the risk of cancer relapse and enhancing overall outcomes, is referred to as adjuvant therapy.

Adjuvant therapies refer to treatments given following breast cancer surgery to reduce the mortality risk by 20–30% [5]. These treatment modalities encompass a range of approaches such as chemotherapy, radiation therapy, hormonal therapy, and targeted therapy, all tailored to the unique characteristics of each patient [6]. Although essential for effective intervention [7], surgery can impact the quality of life for women with breast cancer [8, 9]. A recent study revealed that surgery can lead to poor quality of life, negative body image, and unmet needs, leading to an impaired sense of life satisfaction [10]. To alleviate these challenges, women often seek information from healthcare professionals and rely on various sources of information throughout their courageous battle against cancer [11, 12].

Extensive research has been conducted on the information needs of women facing breast cancer under various circumstances, including during diagnosis, throughout treatment, and at different stages of the cancer journey [13–18]. Typical information needed by the women includes the possibility of a cure, available treatment options, the inherent nature of the disease, and the ongoing prognosis. However, these studies have consistently identified unmet informational needs among this demographic. Factors such as age, education level, type of treatment, and language barriers with healthcare providers may contribute to these unmet needs [19–22].

The information needs of women with breast cancer vary depending on their sociodemographic and clinical characteristics. Notably, younger women tend to exhibit a heightened interest in the preservation of their sexual appeal following diagnosis, whereas older women tend to prioritize self-care practices. Women who have experienced recurrent instances of breast cancer often harbor concerns regarding the potential risks for their family members. Conversely, those who are recently diagnosed or currently undergoing treatment have tendency to express apprehensions regarding the stage of their illness and the likelihood of achieving a cure [16].

Upon receiving a breast cancer diagnosis, women are presented with an array of informational resources from which they can draw upon. Research suggests that their preferred sources of information are healthcare providers, followed by media outlets such as radio and television [23–25]. Notably, a study conducted in South Africa revealed that women primarily acquired information about breast cancer through pamphlets, television, radio, healthcare professionals, as well as personal connections with friends or relatives who had previously encountered the disease [26]. However, participants in the same study acknowledged a dearth of comprehensive understanding regarding breast cancer beyond its mere terminology, highlighting their limited awareness. In addition, women with breast cancer placed great value on practical and experiential insights from fellow patients undergoing similar circumstances [27].

In Ethiopia, primary hospitals with general surgeons provide surgical treatment options for breast cancer patients [28]. Yet, there is a notable lack of research investigating the specific information needs and sources of such information that these women require after undergoing surgery. Addressing these needs has the potential to enhance their overall quality of life [29] facilitate rehabilitation planning [21], and assist in stress management [22]. Moreover, it upholds patients’ rights to receive pertinent information regarding their illness [30]. The objective of this study is to identify the factors determining the information needs of women with breast cancer during various adjuvant therapies.

## Methods

### Study area, period, and design

A facility-based cross-sectional study was conducted among women with breast cancer undergoing several adjuvant therapies at Tikur Anbessa Specialized Hospital (TASH) in Addis Ababa, Ethiopia, from June to August 2022.

TASH, with 700 beds, is a leading cancer treatment facility in Ethiopia, providing comprehensive cancer management services and training for medical students, nurses, dentists, and pharmacists to address community health issues nationwide [31]. Since its establishment in 1997 by the Ethiopian government and the International Atomic Energy Agency, TASH’s oncology unit has been the primary cancer referral center in the country. It comprises an outpatient center for new and follow-up patients and a 19-bed inpatient unit for breast cancer hospitalization [32].

### Study variables

#### Dependent variables

Information needs of women with breast cancer receiving adjuvant therapy, preferred sources of information, and time of access to the information.

#### Independent variables

Sociodemographic variables: age, residency, level of education, occupation, marital status, and sources of treatment expenses.

Clinical variables: stage of the cancer, time since diagnosis, type of surgery, type of treatment, and combination of chemotherapy.

#### Sample size

This study lacks a comprehensive explanation of the methodology used to determine the sample size. Our sample consisted of women receiving adjuvant therapy who were undergoing treatment at TASH. Due to various constraints, including limited budget and time, as well as logistical difficulties, it was not possible to recruit a larger sample size that would meet the standard statistical power requirements. Moreover, the occurrence of women undergoing adjuvant therapy for breast cancer is relatively small compared to the overall population of women with the disease in TASH hospital. Consequently, the expected effect sizes may be extremely small, posing challenges in achieving the desired levels of statistical power even with a larger sample size. As a result, the authors made the decision to include data from all available participants within this limited population rather than striving for statistical power. Additionally, the authors faced difficulties in implementing random sampling due to the nature of the study participants, which further justified the inclusion of all eligible participants.

Out of the 122 participants approached, complete data was obtained from 121 participants throughout the study period.

### Inclusion and exclusion criteria

#### Inclusion criteria

Women who have had at least one cycle of an adjuvant therapy were included as study participants.

Women who are above 18 years of age and can communicate their needs independently without family intervention.

#### Exclusion criteria

Excluded were women with other chronic illnesses and those unable to communicate due to critical illness.

### Data collection procedures

The data collection involved three data collectors and one supervisor, specifically chosen based on their possession of a BSc in nursing, absence of any affiliation with the TASH cancer center, and prior experience in data collection. The data were gathered from both medical cards and directly from the patients. Clinical variables were acquired from medical records, while healthcare providers were consulted for better understanding and clarification of medical terms such as stage of the cancer and chemotherapy combinations.

### Data collection tool

The authors used structured questionnaires adapted from several works of literature to assess the women’s sociodemographic data and clinical information [19–22]. A pretested Toronto information needs questionnaire for breast cancer (TINQ-BC) [33] was used to measure the information needs of women with breast cancer undergoing adjuvant therapies.

### Toronto informational needs questionnaire for breast cancer (TINQ-BC)

The TINQ-BC was originally designed to assess the information needs of women undergoing different breast cancer treatments, including surgery, chemotherapy, and radiation therapy [13]. It consists of 52 items and measures five subscales of information needs: disease, treatment, physical, investigative tests, and psychosocial. The scores range from 52 to 260, with higher mean scores indicating a greater need for information [34]. The assessment uses a five-point Likert scale, ranging from 1 (not important) to 5 (extremely important).

The tool TINQ-BC was first used by Galloway S [33] to explore information needs related to breast cancer among women diagnosed with breast cancer. Then, it was used in Toronto [35] (Cronbach’s alpha (α) of 0.94), Korea (α = 0.97) [36], Egypt (α = 0.97) [37]^,^ and Egypt (α = 0.96) [38].

### Data management quality control

The initial step in validating the TINQ-BC tool involved its translation from its original language, English, into Amharic by a skilled Amharic experts. Subsequently, the translated tool was then translated back into English by proficient English experts and oncologists. This process was undertaken to ensure consistency and confirm the equivalence of meanings. Following the translation process, a pre-test was conducted on a group of 20 women at St. Paul’s Hospital Millennium Medical College, using the translated Amharic version of the tool. Valuable feedback was obtained regarding the clarity of the language used in the tool. Based on this feedback, necessary modifications were made to enhance language clarity. Additionally, the internal consistency of the tool was assessed using Cronbach’s alpha coefficient, which yielded a high value of 0.96. This reinforces the reliability of the tool and further validates its effectiveness. Data collection included reviewing questionnaires for completeness, missing values, and improbable responses.

### Data processing and analysis

Data was collected using Kobo ToolBox and later exported to SPSS (version 25) for analysis. After cleaning outliers and missing values, we conducted various statistical analyses, including re-coding, categorizing, and computing. The independent and dependent variables were analyzed using descriptive statistics (means, standard deviations, frequencies, and percentages) and presented in tables and frequencies.

We conducted a linear regression analysis to determine predictors of information needs. Simple linear regression helped identify candidate variables for multiple linear regression, selecting those with a p-value ≤ 0.25. In the multiple linear regression, variables with p-values ≤ 0.05 were considered statistically significant predictors. The authors described the strength of the relationship between the independent variables using unstandardized β, with a 95% confidence interval (CI). They constructed the final fitted model by forced entry through multiple linear regression analysis methods. Multicollinearity was checked by examining the variance inflation factors (VIF), and it showed no multicollinearity in the final model: the value for each variable was less than ten.

### Result

The age range of the participating women was 23–79 years, with a mean age of 42.45 ± 11.47. Among the participants, 84 (69.4%) were urban dwellers. Of the 121 participants, 61 (50.0%) had attended at least high school. More than half of the participants (73, 60.3%) were unemployed. The majority of participants, 71 (58.7%), were married, and 81 (66.9%) funded their healthcare through insurance. Table 1.

**Table 1:** Sociodemographic characteristics of women with breast cancer on adjuvant therapy at TASH, Addis Ababa, Ethiopia, June to August 2022 (n = 121)

Variables	Frequency	Percentage	Standardized Mean of Information needs (SD)
of Age Distribution			
18–28 29–39 40–50 >50	8 45 45 24	6.6 36.4 37.2 19.8	3.68(0.61) 3.84(0.60) 3.79(0.61) 3.63(0.56)
Mean (SD)	42.45(11.47)		
Residency			
Urban Rural	84 37	69.4 30.6	3.80(0.60) 3.71(0.59)
Level of Education			
No formal education ≤High school ≥ High School	17 43 61	14.0 35.5 50.5	3.47(0.53) 3.85(0.62) 3.61(0.58)
Occupation			
Employed Unemployed	48 73	39.7 60.3	3.77(0.58) 3.76(0.61)
Marital Status			
Never Married Married Separated	14 71 36	11.5 58.7 29.8	3.78(0.45) 3.85(0.62) 3.61(0.58)
Source of treatment expenses			
Sponsored Not Sponsored	81 40	66.9 33.1	3.72(0.56) 3.86(0.65)

### Clinical characteristics of the participants

The majority of the participants were diagnosed with stage II and IV breast cancer: 39 (32.2%) and 38 (31.4%), respectively, while only 8 (6.6%) were diagnosed with stage I breast cancer. As high as 82 (67.8%) received a diagnosis within less than a year. Among the women, only 10 (8.3%) had breast-conserving surgery. Most participants (92, 76%) received a single treatment (chemotherapy, radiotherapy, or hormonal therapy), with AC-T (Adriamycin and Cyclophosphamide, Taxol) being the most common chemotherapy combination used (79, 65.3%). Table 2.

**Table 2:** Clinical Characteristics of Women with Breast Cancer on Adjuvant Therapy at TASH, Addis Ababa, Ethiopia, June to August 2022 (n = 121)

Variables	Frequency	Percentage	Standardized Mean of Information needs (SD)
Stage			
Stage I Stage II Stage III Stage IV	8 39 36 38	6.6 32.2 29.8 31.4	3.72(0.80) 3.78(0.48) 3.73(0.70) 3.81(0.57)
Time Since Diagnosis			
<1year 1-2year 2-3years 3-5years >5years	82 17 9 9 4	67.8 14.0 7.4 7.4 3.4	3.77(0.58) 3.95(0.71) 3.83(0.58) 3.60(0.50) 3.30(0.17)
Type of Surgery			
BCS Mastectomy	10 111	8.3 91.7	3.45(0.65) 3.80(0.58)
Type of Treatment			
Single Treatment Combined Treatment	92 29	76 24	3.82(0.57) 3.60(0.63)
Combination of chemotherapy			
AC AC-T Other	9 79 33	7.4 65.3 27.3	4.00(0.57) 3.75(0.60) 3.74(0.57)

**Note**: BCS: Breast Conservative Surgery; AC: Adriamycin and Cyclophosphamide, AC-T: Adriamycin and Cyclophosphamide, and Taxol, Other: Anastrazole, Cyclophosphamide and docetaxel, Single agent docetaxol, Single agent gemcitabine tamoxifen and trastuzumab

### Descriptive statistics of information needs among breast cancer patients

The total mean of the overall information needs in this study was 194.30 (SD = 28.01), with a range scale of 142–260 and a standardized mean score of 3.74.

Approximately eight items had a mean score above 4.00, including “**How to know if the cancer has come back**” (M = 4.55 with a range scale of 3–5), “**If there is cancer anywhere else in my body**” (M = 4.36 with a range scale of 1–5), and “**Which foods I can or cannot eat**” (M = 4.30 with a range scale of 2–5). The items with the lowest mean scores when compared to others were “**If I can wear brassieres**” (M = 3.07 with a range scale of 1–5) and “**What to do if I become uncomfortable in social situations?**“ (M = 3.42 with a range scale of 1–5). Table 3.

**Table 3:** Descriptive statistics of information needs of women with breast cancer receiving adjuvant therapy at TASH, Addis Ababa Ethiopia, June to August 2022. Items with Highest Score (> 4.00)

Descriptive Statistics					
	N	Minimum	Maximum	Mean	Std. Deviation
If the breast cancer will come back	121	1	5	4.11	0.973
How breast cancer acts in the body	121	1	5	4.05	0.855
If there is cancer anywhere else in my body	121	1	5	4.36	0.856
If it is known what causes breast cancer	121	2	5	4.02	0.861
How to care for my wound or incision	121	2	5	4.12	0.862
How to know if the cancer has come back	121	3	5	4.55	0.658
Which foods I can or cannot eat	121	2	5	4.30	0.813
If my illness is hereditary	121	1	5	4.19	0.969

### Information needs on Disease

The disease subscale incorporates nine items that assess information needs about the disease’s nature, progress, and outcome. The disease subscale was rated between “very important and extremely important,“ with a standardized mean score of 4.00 (M = 36.05 (4.87), on a scale range of 22–45). “**The medical name for my type of breast cancer**” (3.51) was the disease-related information that was least needed, whereas “**how to know if the cancer has come back**” (4.55) was the most crucial component of information.

### Information needs on treatment

This subscale contains 16 items that assess information needs concerning various cancer treatments, how they operate, sensations that may be felt, and their potential adverse effects. This subscale’s overall mean was M = 60.28 (9.49) on a scale of 39–80, with a standardized mean score of 3.77, ranging from “Important to Very Important”. The most overlooked treatment-related information was “**how the treatment will change the way I look**” (3.63), while the most relevant information was “**how the treatment works against the cancer**” (3.98).

### Information needs on investigative tests

This 8-item subscale measures information needs regarding the methods used to assess the severity of the illnesses, their approaches and rationales, and any feelings that may be experienced. With a standardized mean score of 3.65 (M = 29.26 (4.58), on a scale from 21 to 40), information needed on the investigative tests for this study was classified as “important to very important”. “**How I feel during the test**” (3.46) was the least important piece of information, while “**When to have a mammogram**” (3.90) was the most important piece of information.

### Information needs on physical

The needs for preventative, restorative, and maintenance care as a result of the illness and available treatments are incorporated in the physical information needs subscale. It has 11 items. The standardized mean for the physical subscale was 3.65 (M = 40.18 (6.62) on a scale of 27–55). It was deemed “important to very important”. Among the physical information, “**If I can wear a brassiere**” (3.07) was the least needed, while “**Which foods I can or cannot eat**” (4.30) was the most needed.

#### Information needs on psychosocial

The eight-item subscale for psychosocial information needs assesses information needs regarding how to handle the patients’ or their relatives’ feelings. It was itemized as “important to very important” (M = 28.5 (5.19) on a scale of 17–40), and the standardized mean of this subscale was 3.65. For information about psychosocial issues, the least needed information was “**What to do if I feel uncomfortable in social situations**” (3.42), while the most needed information was “**Where my family can go if they need help dealing with my illness**” (3.90).

### Preferable source and time to have information

Regarding preferable sources of information, health professionals were mentioned as a preferred source of information by nearly all women, 115 (95.0%). The least preferable source of information was layman, 14 (11.6%). More than half of the participants preferred to have information before the beginning of the treatments, followed by in the middle of the treatments, at 82 (67.8%) and 28 (23.1%), respectively. Table 4.

**Table 4:** Preferable source and time of information among breast cancer patients following adjuvant therapy at TASH Addis Ababa Ethiopia, June to August 2022 (n = 121)

Variables	Prevalence	Frequency (%)
Preferable source of information		
Health Professionals Layman Media Internet	115 14 51 25	95.0% 11.6% 42.1 20.7%
Preferable time to have information		
Before begging of treatment In the middle of treatment After Treatment Anytime	82 28 24 1	67.8% 23.1% 19.8% 0.8%

#### Predictors of information among women with breast cancer receiving adjuvant therapy

We performed a simple linear regression analysis to examine the relationship between each independent variable and the information needed to select variables in multiple linear regression. We selected the level of education (*P* = 0.044), time since diagnosis (*P* = 0.049), and type of treatment (*P* = 0.032).Similarly, age (*P* = 0.117), source of treatment expenses (*P* = 0.158), and type of surgery (*P* = 0.097) had a P value ≤ 0.25 and were considered for further analysis.

Finally, ten variables were included in the multiple linear regression analysis. Type of treatment and level of education were significant predictors of information needs among women undergoing adjuvant therapy for breast cancer. The model accounted for 18.1% of the variation, with the remaining factors explaining 81.9% (R-square = 0.181, unadjusted R-square = 0.082).

There is a significant positive linear association between the type of treatment, level of education, and information needs. When all other variables in the model were held constant, women with single treatment options were 12.68 times more likely to have information needs when compared with those who are on combined treatment options, 12.68 (95% CI=(0.68, 24.68); *P* = 0.039). The results also showed that women who had completed higher education or above were 17.8 times more likely to have information needs than those with no formal education (17.1 (95% CI=(1.47, 34.14); *P* = 0.033). Table 5.

**Table 5:** Multiple linear regression analysis predicting information nee of women with breast cancer on adjuvant therapy at TASH, Addis Ababa, Ethiopia, June to August 2022 (n = 121)

Predictors	Crud Unstandardized ß 95% CI of β	Adjusted Unstandardized ß 95% CI of β
Age Time Since Diagnosis	-0.35(-0.79,0.09)** -3.26(-6.61, 0.02)**	-0.169(-0.643, 0.305) -3.28(-7.3, 0.74)
Residence		
Rural	**Ref**	
Urban	-3.78(-7.19, 14.74)	0.31(-10.78, 11.4)
Occupation		
Employed	**Ref**	
Unemployed	1.67(-8.68, 12.01)	8.52(-2.45, 19.49)
Marital Status		
Never Married Married Separated	**Ref** 5.04(-11.16, 21.23) -3.65(-21.09, 13.8)	3.66(-12.8, 20.12) -1.78(-20.06, 16.5)
Level of Education		
No formal education	**Ref**	
<High School ≥High School	10.59(-5.02, 26.20) 18.32(3.39, 33.27)*	9.42(-6.59, 25.42) 17.81(1.47, 34.14)*
Source of Funding		
Not Sponsored	**Ref**	
Sponsored	-7.66(-18.33, 3.01)**	-2.84(-14.51, 8.84)
Combination of Therapy		
AC-T AC	2.40(-9.14, 13.95) 10.61(-10.33, 31.56)	0.33(-13.54, 14.21) 9(-13.45, 31.45)
Others	**Ref**	
Type of Surgery		
Mastectomy	15.37(-2.81, 33.55)**	12.92(-6.21,32.04)
BCS	**Ref**	
Type of Treatment		
Single treatment	12.77(1.14, 24.41)*	12.68(0.68, 24.68)*
Combined treatment	**Ref**	

**Notes**: Constant = 166.4, R = 0.425, R Square = 0.181, Adjusted R Square = 0.082; **Significant at p value < 0.25, * Significance value < 0.05. Dependent variable: Information need. Max VIF 1.22 (no Multicollinearity: VIF < 10)

## Discussion

Effective communication can lessen patients’ anxiety about learning devastating news related to their illness, leading to a substantial improvement in their quality of life [23, 39, 40], and it can also allow a change in the patient’s disease trajectory to improve their well-being and its persistence until the end of life [11, 19, 41, 42].

Based on the available literature, providing information to women with breast cancer who have undergone surgery as part of their adjuvant therapy has been shown to improve their quality of life [5, 43–47]. However, there is a lack of research on the information needs of women with breast cancer after surgery in Ethiopia. Therefore, the objective of this study was to identify the factors influencing the information needs of women undergoing various adjuvant therapies for breast cancer. The study also emphasizes the sources of information and the optimal timing for providing it.

The mean total information needs of women with breast cancer in our study were 194.30 (28.01) on a scale ranging from 142 to 260. This score is lower than that reported in other studies [48–52]. There are several possible reasons for this variation, such as the fact that our participants were undergoing adjuvant therapy, the smaller sample size, low health literacy, and limited access to information. Interestingly, our findings were even lower than a previous study in Ethiopia, which included women with breast cancer in general (mean score of 238.7, 22.50) [13]. This difference may attributed to the smaller sample size, the specific characteristics of our study participants, and the cross-sectional study design.

In our study, there was a high demand for disease-related information, followed by treatment-related information, which aligns with findings from other studies [13, 33, 35, 36, 52, 53]. Women undergoing adjuvant therapy for breast cancer expressed a strong need for information on topics such as detecting cancer recurrence, the presence of cancer in other parts of the body, and the hereditary nature of the illness [13, 33, 53, 54]. This consistent pattern suggests that regardless of the specific treatment and intervention, breast cancer patients are concerned about the possibility of cancer returning, metastasis, and genetic predisposition. The unpredictable nature of the disease, its psychological impact, and the potentially life-threatening side effects contribute to this information gap. Consequently, the women are compelled to establish a strong rapport with healthcare providers to gain knowledge about their prognosis, understand the treatment’s effects, manage side effects, and develop coping strategies [55, 56].

In other studies, there was an increased demand for investigative test-related information followed by treatment-related information [54, 57]. Similarly, in a study focusing on women receiving radiotherapy, there was a higher need for disease-related information in addition to information about investigative tests for cancer [35]. The difference in study populations and hospital settings may contribute to this variation. In countries like Ethiopia, women express concerns about the outcome of the disease, and the costly treatments they are undergoing. Since the hospital is responsible for most investigative procedures, women are less concerned about the investigation process despite long waiting lists.

The women could also wonder about information on sexuality, daily life activities, emotional support, how to prevent breast cancer, self-care, dietary follow up, etc. [52, 58, 59]. However, there exists a meticulously designed dietary regimen that can intricately contribute to the prevention of breast cancer by effectively stabilizing gene mutations [60]. Moreover, in Ethiopia, women are embarrassed to discuss their sexual lives, and seek emotional assistance because they are supposed to be strong in all circumstances to establish a stable family. In general, a systematic review [50] reported the prognosis of the disease, diagnosis, and treatment options for the disease as the main areas where information is needed.

The study found that sociodemographic and clinical factors can influence women’s information needs, unlike other studies [13, 36, 61, 62], no significant differences were observed based on age group, cancer stage, and area of residency. This lack of variation may be due to the majority of participants falling within the same age group and small sample size. However, there were significant differences in information needs based on education level [61]. Women with higher educational level tended to have higher information needs, likely because they have greater knowledge exposure and actively seek more information. Additionally, the study found significant differences in information needs based on the type of treatment [51], with women on single treatments expressing higher information needs than those on combined treatments. This may attributed to the perception that combined treatments provide more comprehensive care. Thus, they neglect the need for information.

In the current study, the preferred source of information was health professionals (n = 115, 95.0%), followed by media (n = 51, 42.1%), which is congruent with the findings of previous studies from Ethiopia [13, 63]. Justifications provided for such preferences were issues concerning media not giving due attention to providing information regarding breast cancer and limited access to the internet. As a result, patients prefer health professionals and women with the same experience as their main source of information [13].

The preferred time for information in the current study was ‘before the initiation of treatments’ in 82 patients (67.8%), while ‘any time’ was the least preferable time for information delivery in 1 patient (0.8%). This was similar to several studies [13, 36]. This contributes to the need for women to commence an informed treatment process. Moreover, getting information prior to starting treatments helps the women to get themselves psychologically prepared since it will enable them to manage the side effects of treatments, which in turn improve their quality of life. Because, even the advanced cancer treatment has mounted threat to fertility. Thus, the women needs to get counseled on availability of safe fertility preservation [64, 65].

The optimal period for dispensing information in the present investigation was determined to be “before the commencement of treatments” for a majority of 82 patients (67. 8%), whereas only one patient (0.8%) exhibited a preference for receiving information “at any time.“ This aligns with previous studies [13, 36]. This contributes to the need for women to commence an informed treatment process. Moreover, getting information prior to starting treatments helps the women to get themselves psychologically prepared since it will enable them to manage the side effects of treatments, which in turn improve their quality of life. Furthermore, considering that advanced cancer treatments present significant threats to fertility, women must be counseled regarding the available options for safe fertility preservation [64, 65].

This study has a limitation in that it is a cross-sectional study, which only provides insight into the information needs of women during a specific time period. Therefore, we cannot determine how these needs may change at different times of the disease process. Additionally, the small sample size of women on adjuvant therapy may impact the generalizability of the findings. To improve care and enhance the quality of life for women with breast cancer, future research should consider studying women in different treatment groups from multiple center, such as those on neoadjuvant therapy, radiotherapy alone, and post-surgery before starting adjuvant therapy. This would provide a more comprehensive understanding of their information needs and enable tailored support accordingly.

## Conclusions

The women had high overall information needs. The highest information needs were observed in the disease and treatment domain. Disease recurrence, its spreading tendency and familial aspects were the most needed individual information. Thus, equipping health professionals with the ability to deliver the most sought-after information would aid in improving women’s quality of life.

Health care professionals were discovered to be the most important source of information, followed by the media (radio and television). In light of this, health care professionals should equip themselves and develop strong bonds with the women to provide gratifying information and get trusted. Women also need to have written and orally delivered information according to their education level about the treatment prior to its commencement; hence, they would have a better prognosis with better controlled side effects.

Additionally, care for women with breast cancer should include the use of media to disseminate information. Additionally, creating a space where the ladies may talk freely to one another and be accessible to one another would be helpful. A study has proven the effectiveness of media and practical information from a person with a very similar condition [26].

## Data Availability

The datasets used and analyzed in the current study are available upon reasonable request from the corresponding author.

## Abbreviations

QOL: Quality of life
TASH: Tikur Anbessa Specialized Hospital
TINQ-BC: Toronto information needs questionnaire for breast cancer
VIF: Variance inflation factors
